# Correlation between the In-Plane Critical Behavior and Out-of-Plane Interaction of Ternary Lipid Membranes

**DOI:** 10.3390/membranes13010006

**Published:** 2022-12-21

**Authors:** Ting Hsuan Ko, Yi-Fan Chen

**Affiliations:** Department of Chemical and Materials Engineering, National Central University, Taoyuan 32001, Taiwan

**Keywords:** critical fluctuations, membrane fusion, liquid-liquid phase separation, 2-D Ising model

## Abstract

Liquid-liquid phase-separating lipid membranes belong to the 2-D Ising universality class. While their in-plane critical behaviors are well studied, how the behaviors modulate out-of-plane interactions is rarely explored, despite its profound implications for biomembranes and 2-D ferromagnets. Here, we examine how the interlayer interaction, manifested as membrane fusion, is affected by the membranes’ critical fluctuations. Remarkably, the critical fluctuations suppress membrane fusion, suggesting a correlation between critical behaviors and interlayer interactions for 2-D Ising systems.

## 1. Introduction

**Figure 1:**
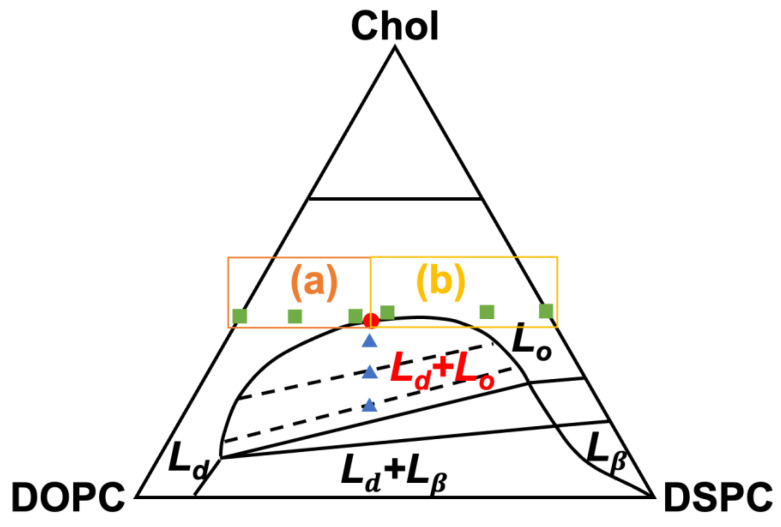
Schematic of the ternary phase diagram for the lipid membranes of DOPC/DSPC/cholesterol (Chol) at 22 °C, based on ref. [13]. *L_d_*, *L_o_*, and *L_β_* are the liquid-disordered, liquid-ordered, and solid-gel phases, respectively, for the in-plane organization of the membranes. The filled circle is the miscibility critical point. The compositions of the homogeneous and laterally heterogeneous membranes are marked by filled squares and triangles, respectively. Rectangles mark two compositional regimes, (a) and (b), where the fusion extent of the homogeneous membranes behaved distinctly.

Ternary lipid membranes composed of a low melting-temperature lipid, a high melting-temperature lipid, and cholesterol (Chol) are 2-dimensional (2-D) entities capable of exhibiting liquid-liquid phase separation and critical phenomena. At specific compositions and temperatures, the membranes are laterally heterogeneous and phase-separate into coexisting liquid phases, the liquid-disordered (*L_d_*) and liquid-ordered (*L_o_*) phases [1,2]. The compositional distinction between the phases, conventionally denoted with tie lines in the dome-shaped region of a ternary phase diagram (Figure 1), becomes obscure and their line tension diminishes when a specific overall composition for such a membrane is approached at an appropriate temperature. In approaching this composition, known as the miscibility critical point, the correlation length of the membranes is also diverging when critical phenomena emerge [3]. Even when the membranes are laterally homogeneous, the critical point still perturbs the membranes as long as they are compositionally near the critical point. In these conditions, local fluctuations in density or composition occur and spawn short-lived domains in the otherwise homogeneous membranes. This phenomenon is known as critical fluctuations and is commonly observed in entities showing critical behaviors [4,5]. The compositional proximity to the critical point dictates the extent of the fluctuations for the homogeneous membranes, as the correlation length diverges upon the approach of the critical point [3,6]. In addition to varying with composition, the correlation length, ξ, also varies with temperature for an appropriate composition, following power laws of the form, $\xi \sim {\left[\left(T-T_{c}\right)/T_{c}\right]}^{-\nu }$, where $T_{c}$ is the critical temperature and ν is the critical exponent for ξ [5]. Systems of diverse natures may exhibit identical values for the critical exponents, and those showing identical critical-exponent values belong to the same universality class. For the ternary lipid membranes, ν was measured to be ~1 [3], placing the membranes to the universality class of the 2-D Ising model, where each 2-D lattice point can only assume one of two states (e.g., up- and down-spin).

That lipid membranes can be prepared as unilamellar vesicles (ULVs), a hollow spherical structure bound by a single lipid membrane, and free of substrates renders the ternary lipid membranes a rare 2-D Ising system capable of interacting with one another in a 3-D space via fusion. Lipid membrane fusion is a biologically relevant process, through which two ULVs fuse their membranes, mix their contents and merge into a single ULV [7,8]. The physical properties of a lipid membrane determine how readily it can fuse with another. Earlier studies have shown that lipid membranes exhibiting a stronger tendency of curving and stronger resistance to bending fuse more readily [9]. Besides, the phase behavior in the membrane plane is also crucial: Liquid-liquid phase separation facilitates fusion, which is attributed to the presence of the *L_d_* − *L_o_* phase boundaries [10,11,12]. In this context, an interesting question may naturally arise: Can the transient domains spawned from the critical fluctuations and the transient phase boundaries thereof contribute to membrane fusion? Or, in another context, can the in-plane critical fluctuations occurring in the 2-D Ising entities modulate their interactions with one another in 3-D space?

The question is not trivial. Biologically, cell membranes are compositionally near their critical points and therefore exhibit critical fluctuations in physiological conditions [14,15]. The critical fluctuations in cell membranes have been suspected as the molecular mechanism underlying the lipid rafts [6,15,16], transient nanoscale domains enriched in Chol, and responsible for cellular functions such as signal transduction [17,18]. It is therefore not inconceivable that the critical fluctuations are involved in other biological processes, particularly membrane fusion, which, among others, underlies viral entry and synaptic neurotransmissions [7,8], given the aforementioned influence on the fusion of domain boundaries. Accordingly, cells might exploit the critical fluctuations to regulate membrane fusion and the downstream cellular events; exploring the underlying mechanisms will advance our understanding of cellular machinery and ultimately lead to therapeutics for related diseases. On the other hand, discussions on 2-D Ising systems have focused on the in-plane phase behaviors and seldom on how 2-D Ising entities interact with one another and how these interactions are possibly associated with the critical behaviors. However, for the Ising systems showing interlayer interactions, such as the stacks of 2-D van der Waals ferromagnets [19,20], these in-plane phase behaviors might have 3-D implications: They might modulate or be modulated by the interlayer interactions, which in turn dictates the magnetic properties of the 2-D ferromagnet stacks. Therefore, exploring how the critical fluctuations perturb the interlayer interactions and investigating whether such a correlation is universal among the diverse constituents of the 2-D Ising universality class is not only scientifically intriguing but also bears technological significance. In this context, the ULVs whose compositions are near the critical point and whose critical temperatures are around room temperature may serve as an accessible platform for such investigations. Remarkably, our findings suggest that the in-plane critical behavior of the lipid membranes can modulate the intermembrane interaction. This may provide a starting point for the investigations on other 2-D Ising systems.

## 2. Materials and Methods

### 2.1. Materials

Lipids, DSPC (distearoylphosphatidylcholine or di18:0PC; cat. no. 850365C), DOPC (dioleoylphosphatidylcholine or di18:1PC; cat. no. 850375C) and Chol (cholesterol; cat. no. 700000P), were sourced from Avanti Polar Lipids and used as received. Fluorescence dyes, DPA (dipicolinic acid; cat. no. AAA1226314), and TbCl_3_ (terbium(III) chloride hexahydrate; cat. no. AC199610050) were purchased from Thermo Fisher Scientific (Waltham, MA) and also used without further processing. Other chemicals, including HEPES (cat. no. V900477), sodium citrate tribasic dihydrate (cat. no. S4641), PEG 8000 [poly(ethylene glycol); cat. no. P2139] and EDTA (ethylenediaminetetraacetic acid; cat. no. E9884), were products of Sigma-Aldrich (St. Louis, MO, USA) and also used as received.

### 2.2. ULV Preparation

Unilamellar vesicles (ULVs) were prepared by extrusion, following an established protocol [21]. DSPC, DOPC, and Chol in desired molar ratios were dissolved and mixed in chloroform. The solution was dried with a gentle nitrogen flow and placed under a vacuum overnight for the complete removal of chloroform. The acquired dry lipid film was resuspended in a 10 mM HEPES buffer (pH = 7.4). The suspension was homogenized by 5 runs of alternate vigorous vortex and freeze-thaw cycles, which resulted in the formation of multilamellar vesicles (MLVs). Extrusion of the MLV solution was carried out with an extruder set from Avanti Polar Lipids (cat. no. 610000), via 200 nm and 50 nm pores consecutively, to obtain ULVs. The final lipid concentration was 2 mg/mL.

ULVs encapsulating fluorescence dyes, DPA or TbCl_3_, were prepared similarly. Here, the HEPES buffer (pH = 7.4) used for resuspension additionally contained 100 mM DPA or 100 mM TbCl_3_ + 100 mM sodium citrate. After the extrusion that produced ULVs, the fluorescence dyes outside of ULVs were removed with a centrifugal concentrator of 30,000 molecule-weight cutoff (cat. no. VS0151, Vivaproducts). Removal of the free dyes was confirmed by measuring the fluorescence of the DPA-encapsulating ULVs in the presence of TbCl_3_ outside of the ULVs and vice versa.

Dynamics light scattering indicated the diameters of the ULVs to be ~70 nm and with a polydispersity index of ~0.2. The solutions were used within 3 days of preparation.

### 2.3. Fusion Assay

Membrane fusion between ULVs of differing compositions was fluorescently examined by employing DPA- and TbCl_3_-encapsulating ULVs in a fusion assay according to an established protocol [22]. Upon completion of membrane fusion, the contents of two ULVs were mixed and DPA inside one was allowed to complex with Tb^3+^ inside the other, which would considerably enhance the fluorescence of Tb^3+^ and thereby quantified the fusion extent. To carry out the assay, DPA-encapsulating ULV solution was mixed with the same amount of TbCl_3_-encapsulating ULVs solution. Subsequently, a 50 wt% PEG 8000 solution of the same volume was added to the ULV solution to osmotically drive membrane fusion. The lipid concentration in the mixture was then adjusted to 0.1 mg/mL with 10 mM HEPES buffer containing 1 mM EDTA, the addition of which was to chelate Tb^3+^ leaking from or otherwise outside the ULVs and thereby suppress complexation between DPA and Tb^3+^ outside the ULVs. Fluorescent intensity at 543 nm (excited at 279 nm) of the DPA-Tb^3+^ complexes formed after membrane fusion were measured after 30-min incubation. The highest possible fluorescent intensity that could result from DPA-Tb^3+^ complexation for a given sample was assessed by lysing the ULVs with 0.01% (*v*/*v*) Triton X-100, allowing all DPA and Tb^3+^ to complex with each other. Fluorescent intensity was also measured for the comparable but PEG-free ULV solution as the background fluorescence in the determination of fusion extent. The fusion extent was quantified as

(1) $$\left(I-I_{0}\right)/\left(I_{max}-I_{0}\right)\times 100\%,$$

where I is the intensity of a given sample after fusion, $I_{0}$ is the background fluorescence determined from its PEG-free counterpart and $I_{max}$ is the maximal intensity measured after the addition of the detergent, Triton X-100. The fusion assay for each lipid composition was repeated thrice with independently prepared samples to ensure the reproducibility of the measurements.

### 2.4. SAXS Measurement on Membrane Thickness

Small-angle X-ray scattering (SAXS) was employed to measure the membrane thickness of ULVs as well as to confirm their unilamellarity. The data were collected from 10 mg/mL ULV solutions with 15 keV X-rays at Beamline 23A of the National Synchrotron Radiation Research Center (Hsinchu, Taiwan). Prior to the formal data collection, different parts of a sample were illuminated with X-rays to compare their scattering profiles. Only when the profiles were identical and free of sharp diffraction peaks and the sample was therefore highly homogeneous and unilamellar would the experiment move to the next stage; otherwise, the sample would be removed from the data collection process. The raw SAXS images collected with the Pilatus 1MF pixel detector were corrected for background and solvent scattering and azimuthally integrated with a custom-made program developed by the beamline to obtain the scattering profiles of scattering intensity $I\left(\left|\vec{Q}\right|\right)$ against $\left|\vec{Q}\right|$, where the magnitude of the momentum transfer $\left|\vec{Q}\right|$ is defined as $\left|\vec{Q}\right|=4\pi \sin\theta /\lambda$, with 2θ the scattering angle and λ the incident X-ray wavelength.

The obtained scattering profiles were then subjected to fitting against a model for the transmembrane electron-density distribution $\rho \left(z\right)$, where z is the transverse distance from the bilayer midplane at z=0, to extract the membrane thickness. For lipid membranes with the bilayer structure, where two single-molecular layers of lipids are arranged back-to-back with their hydrocarbon chains in contact and their hydrophilic headgroups enclosing the hydrophobic core, $\rho \left(z\right)$ can be modeled as [23],

(2) $$\rho \left(z\right)=\exp\left[-\frac{{\left(z-z_{H}\right)}^{2}}{2\sigma_{H}^{2}}\right]+\exp\left[-\frac{{\left(z+z_{H}\right)}^{2}}{2\sigma_{H}^{2}}\right]-\rho_{r}\exp\left[-\frac{z^{2}}{2\sigma_{C}^{2}}\right],$$

where $\rho_{r}=\rho_{C}/\rho_{H}$. Here, the distributions of the headgroups and the methyl group of the hydrocarbon chains are described with Gaussian functions and centered at $z=\pm z_{H}$ with width $\sigma_{H}$, and at z=0 with width $\sigma_{C}$, respectively (Subscripts *H*, *C*, and *r* represent head, chain, and relative, respectively). Fourier-transforming $\rho \left(z\right)$ leads to the form factor $F\left(\vec{Q}\right)$ expressed as,

(3) $$F\left(\vec{Q}\right)={\left(2\pi \right)}^{1/2}\left[2\sigma_{H}\exp\left(-\sigma_{H}^{2}{\left|\vec{Q}\right|}^{2}/2\right)\cos\left(\left|\vec{Q}\right|z_{H}\right)-\sigma_{C}\rho_{r}\exp\left(-\sigma_{C}^{2}{\left|\vec{Q}\right|}^{2}/2\right)\right].$$

$F\left(\vec{Q}\right)$ is subsequently related to the scattering intensity $I\left(\left|\vec{Q}\right|\right)$ via,

(4) $$I\left(\left|\vec{Q}\right|\right)={\left|F\left(\vec{Q}\right)\right|}^{2}/{\left|\vec{Q}\right|}^{2}.$$

**Figure 2:**
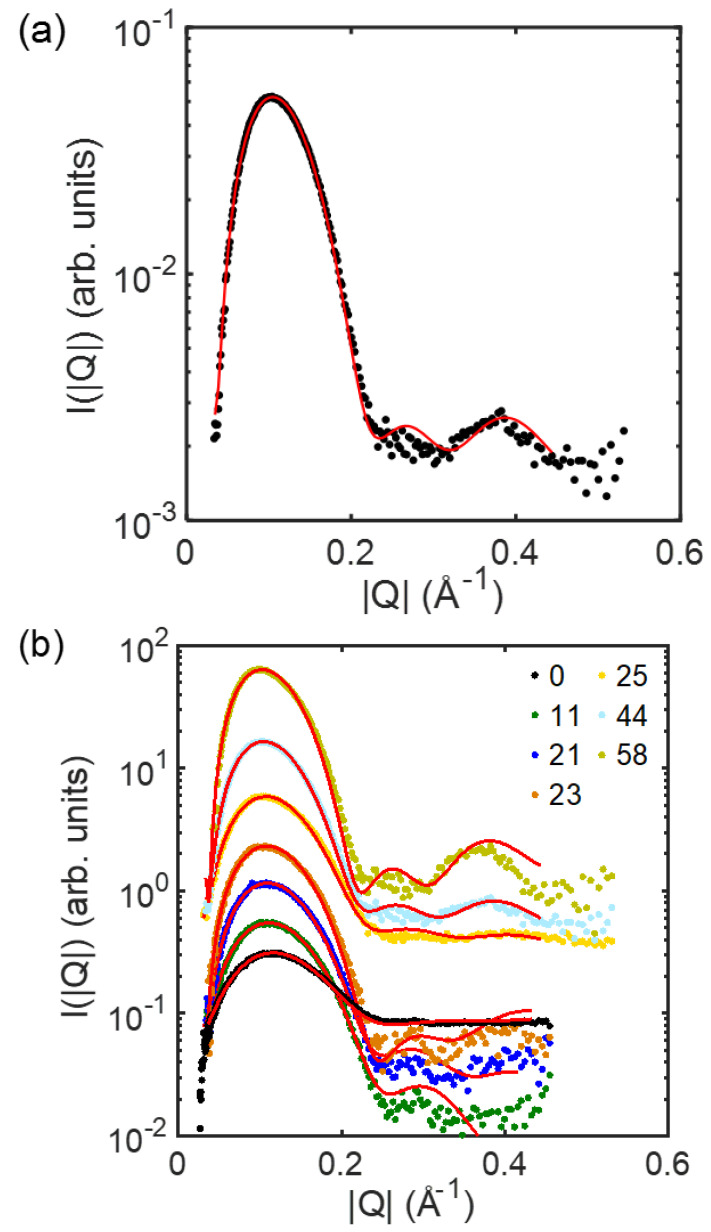
Scattering profile of $I\left(\left|\vec{Q}\right|\right)$ gainst $\left|\vec{Q}\right|$ for the ULVs with (**a**) the composition of DOPC/DSPC/Chol = 14/44/42 and (**b**) all the compositions for homogeneous membranes (designated with the DSPC content; refer to Table 1 for the full compositions) at 23 °C. Solid curve is the fit against Equations (2)−(4). Curves in (**b**) are vertically shifted for clarity.

**Figure 3:**
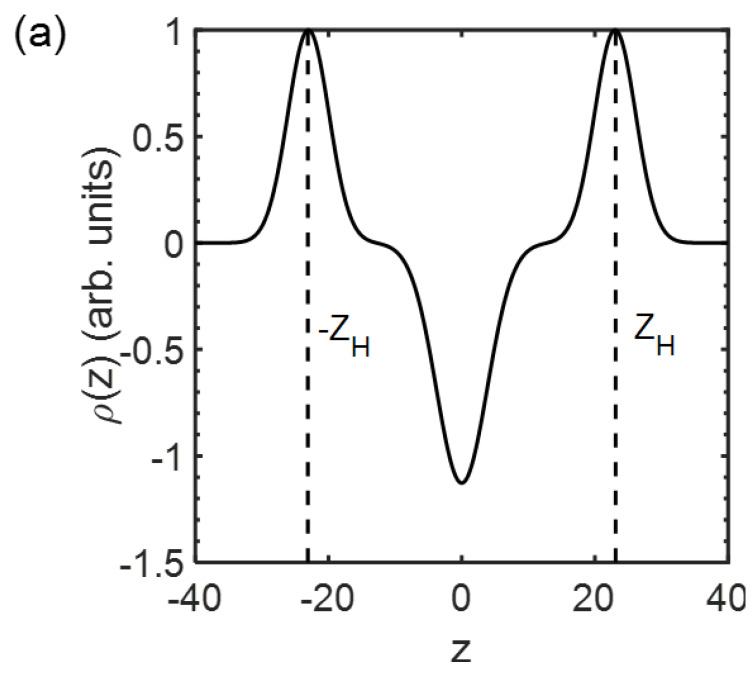

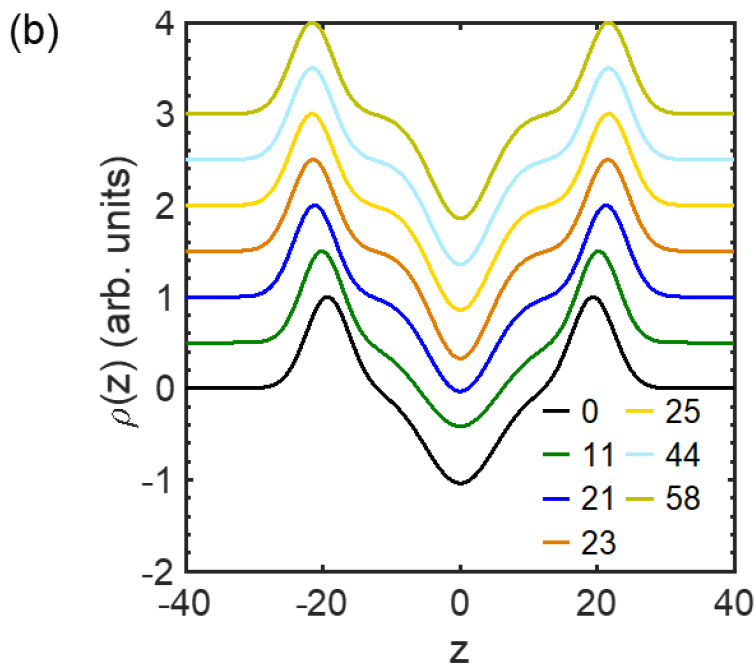
Electron density profile p(z) transverse a lipid membrane extracted from the fit against Equations (2)−(4) in Figure 2 for (**a**) the composition of DOPC/DSPC/Chol = 14/44/42 and (**b**) all the compositions for homogeneous membranes at 23 °C. $z_{H}$ marks the position of the headgroups, and $2z_{H}$ is defined as the membrane thickness. Curves in (**b**) are vertically shifted for clarity.

By fitting the experimentally collected $I\left(\left|\vec{Q}\right|\right)$-versus-$\left|\vec{Q}\right|$ profiles against Equations (2)–(4) (Figure 2), one can obtain $\rho \left(z\right)$ and determine several structural parameters for the membranes, including $\pm z_{H}$ (Figure 3). In this study, the distance between the centers of the distributions of the headgroups, i.e., $2z_{H}$, was treated as the membrane thickness of the ULVs.

## 3. Results and Discussion

To explore the correlation between the critical fluctuations and membrane fusion, ULVs of ~70 nm in diameter were prepared from Chol, DOPC (dioleoylphosphatidylcholine, a low melting-temperature lipid), and DSPC (distearoylphosphatidylcholine, a high melting-temperature lipid). The lipids were mixed in varied molar ratios (Figure 1 & Table 1), in accordance with the published phase diagram for the ternary lipid membranes at 22 °C [13], to make two series of ULVs featuring either laterally heterogeneous membranes or homogeneous membranes exhibiting critical fluctuations. The membranes exhibited varying degrees of compositional proximity to the critical point. For the heterogeneous membranes, the compositions were so selected that they were not only located within the *L_d_* − *L_o_* coexistence region of the phase diagram but also at the midpoints of the tie lines. Thus, the heterogeneous membranes were expected to phase separate into *L_d_* and *L*_o_ of roughly equal areal fractions, regardless of their proximity to the critical point. The homogeneous membranes’ proximity to the critical point was modulated by varying the molar ratios of DSPC and DOPC, with Chol fixed at ~40 mol%. Figure 1 shows the compositional distribution of the adopted ULVs in a schematic phase diagram. It is noted that the ternary lipid membranes of larger (>1 μm) and smaller (~60 nm) ULVs were observed to show comparable critical behaviors [3,24]; the aspects of the critical behavior relevant to this present study are thus considered only marginally dependent on ULV size. Fusion among the ULVs was fluorescently examined at 23 °C by loading the fluorescence dyes, DPA (dipicolinic acid) and TbCl_3_, into separate ULVs. Osmotically driven by PEG 8000, the DPA- and TbCl_3_-encapsulating ULVs fused with one another and mixed their contents, allowing the dyes to complex and enhance Tb^3+^ fluorescence. The fusion extent was quantified by measuring the fluorescence intensity.

**Figure 4:**
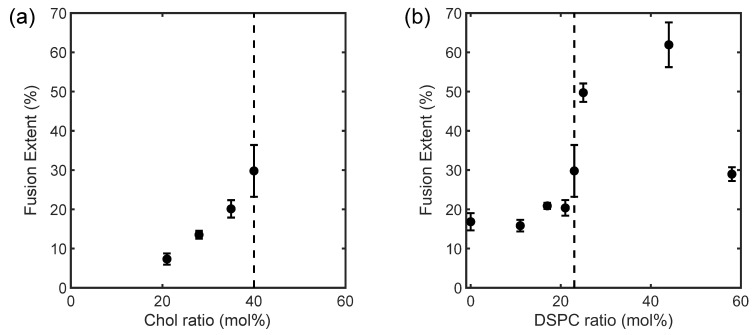
Fusion extent for the (**a**) laterally heterogeneous and (**b**) homogeneous membranes as a function of composition at 23 °C. The compositions are designated by their (**a**) Chol and (**b**) DSPC contents. Detailed compositions are given in Table 1. Dash lines mark the critical composition. The measurements were repeated at least triply with independently prepared samples.

The ULVs with heterogeneous membranes increased their fusion extent monotonically when Chol was raised from 20 to 40 mol% and the critical point was approached from within the coexistence region (Figure 4a). The former trend is consistent with earlier observations that Chol facilitates membrane fusion [9,25], while the latter indicates a potential contribution to fusion from the membranes’ phase behaviors. For the ULVs with homogeneous membranes, fusion extent was essentially unchanged upon raising DSPC, until the critical point (at DSPC = 23 mol%) was reached (Figure 4b). Remarkably, further raising DSPC beyond the critical point gave rise to a drastic increase in fusion extent, before the extent drastically declined when the solid gel *L_β_* phase emerged at DSPC = 60 mol% (i.e., DOPC = 0 mol%). The variation in fusion extent for the homogeneous membranes can therefore be parted into two regimes, separated by the critical point (rectangles in Figure 1).

The fusion assay affirms lipid composition as a key determinant for membrane fusion. The composition’s role here may arise from its influences on both the elastic properties and phase and critical behaviors of a membrane. Regarding membrane elastic properties, Chol’s strong tendency of forming curved structures is known to build up bending strains within a planar membrane enriched in Chol [26]. (For ULVs of 70 nm, the thinness of their membranes, ~5 nm, allows them to be viewed as locally planar.) The strains energetically favor the formation of the highly curved intermediate structures of fusion (e.g., the stalk structure), and fusion is thereby promoted [7]. This partially explains why the heterogeneous membranes were more prone to fuse when Chol was raised (Figure 4a). On the phase-behavior side, raising Chol also drove the heterogeneous membranes toward the critical point. When a heterogeneous membrane approaches its critical point, the compositional difference and thus the line tension between the coexisting *L_d_* and *L_o_* phases diminishes. The reduction in line tension lowers the energetic cost of forming phase boundaries. As a result, the phase domains shrink in size, and the phase boundary propagates [3,24]. A recent computational study suggests the phase boundaries are unique sites for the stalk structure and making it more energetically favorable [12]. Hence, the phase-boundary propagation could favor fusion between the heterogeneous membranes by facilitating the formation of the stalk structure. Collectively, the modulations in the elastic property and phase behavior of the heterogeneous membranes might contribute to the trend in Figure 4a. This understanding also supports the notion that the *L_d_* − *L_o_* phase boundary per se facilitates fusion, in addition to being the preferred site for the fusion-peptide insertion that initiates fusion in viral infections [10,11].

**Figure 5:**
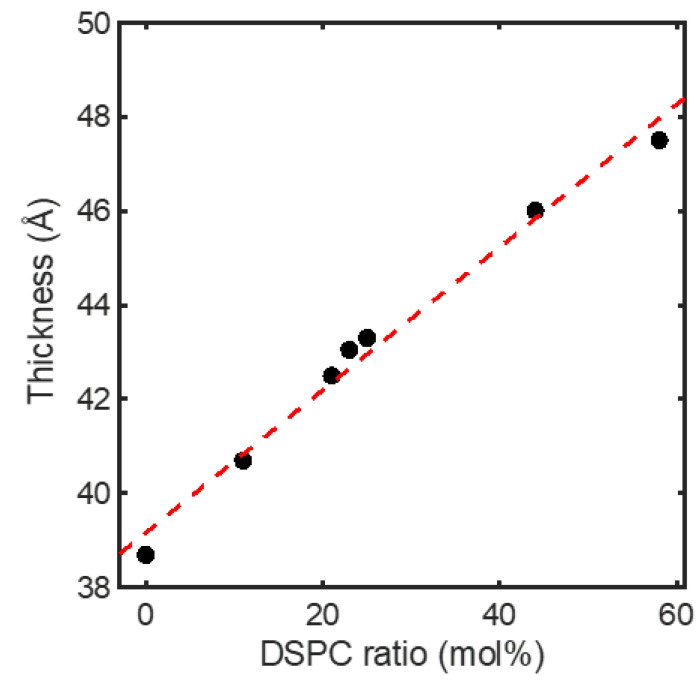
Membrane thickness determined with SAXS for the homogeneous membranes as a function of composition at 23 °C. The compositions are designated by their DSPC contents. Detailed compositions are given in Table 1. Dashed line is a linear fit to guide the eyes.

While approaching the critical point favored fusion between the heterogeneous membranes, a different story emerges when one examines fusion between the homogeneous membranes (Figure 4b). Again, the observed trend for the latter might be ascribed to modulations in elastic property and critical behavior of the membranes. As shown by our small-angle X-ray scattering (SAXS) measurement on the membrane thickness of the ULVs at 23 °C, the homogeneous membranes thickened steadily when DSPC was raised at the expense of DOPC (Figure 5). This thickening reflects membrane rigidification upon incorporating more DSPC in the membranes, as the incorporation of saturated lipids (e.g., DSPC) rigidifies and thickens a membrane by making the hydrocarbon chains of lipids more ordered [27]. Given that rigidifying lipid membranes raises their chance to fuse with one another [22], the homogeneous membranes should have displayed increasingly higher propensities of fusion when DSPC was raised. Instead, the fusion extent did not rise monotonically but exhibited two regimes, where it barely changed [rectangle (a) in Figure 1] or rose considerably [rectangle (b) in Figure 1], when raising DSPC drove the membranes to and away from the critical point (Figure 4b). This suggests that another factor must have contributed to this bimodal variation. Since the extent of the critical fluctuations depends on a membrane’s proximity to the critical point [3,6], the critical fluctuations in the homogeneous membranes would switch from being enhanced to being suppressed when the membranes crossed the critical point. Following this thread, we note that the fusion extent barely changed when the critical fluctuations intensified whereas the surge in fusion occurred when the critical fluctuations subsided. It is therefore tempting to infer this switch is the cause of the bimodal variation. Given that membrane rigidification promotes fusion [22], this further suggests that enhancing and suppressing the critical fluctuations could, respectively, impede and facilitate fusion. Accordingly, enhancing the critical fluctuations and rigidifying the membranes by raising DSPC toward the critical point [following rectangle (a) in Figure 1] led to opposite effects on fusion, which might offset each other and leave the fusion extent unchanged. On the other hand, raising DSPC beyond the critical point [following rectangle (b) in Figure 1] further rigidified the membranes but suppressed the critical fluctuations, likely synergistically promoting fusion and resulting in a surge in fusion. The bimodal variation in fusion would thereby arise. Given that bringing two membranes into close proximity is the first and highest energetic barrier for fusion [28], the critical fluctuations might exert their influences on fusion by perturbing the forces between the two membranes.

The critical fluctuations’ involvement in the fusion-embodied intermembrane interaction has both biological and physical implications. Biologically, this suggests that cells might regulate fusion (e.g., fusion occurs between the membranes of neurons and synaptic vesicles in the secretion of neurotransmitters) via critical fluctuations. Given the drastic variation in the fusion extent upon even a minor compositional change around the critical point (Figure 4b), metabolically modulating the lipid composition of a cell membrane, which is in its critical composition [14,15], constitutes a highly efficient way to regulate fusion. Therefore, maintaining a cell membrane in its critical composition may not only endow a cell with lipid rafts [6,15,16] but also afford it the capability to efficiently regulate fusion. Physically, with their critical temperatures close to room temperature, the ULVs provide a readily accessible platform for investigations on how individual entities from the 2-D Ising universality class interact with one another when they are near the critical point. This turns out to be not trivial. Eyeing the promising potentials of 2-D materials, scientists had long searched for the 2-D version of ferromagnets, but to no avail until very recently [19,20]. (Interestingly, the very first system theoretically constructed for the 2-D Ising universality class is 2-D ferromagnets.). Many intriguing observations have since been reported: The CrI_3_ and CrGeTe_3_ monolayers, the first 2-D ferromagnets discovered, transition to ferromagnets at higher critical temperatures when they are stacked to bilayers or tri-layers [29,30], while the critical temperature for another 2-D ferromagnet, VI_3_, is higher for its monolayers than for its bilayers and tri-layers [31]. For CrI_3_, how many layers are stacked even determines its magnetism, with the monolayers and tri-layers behaving as ferromagnets and the bilayers as antiferromagnets [29]. It can be possible that the critical behaviors of the 2-D ferromagnets are associated with the interlayer interaction responsible for their anomalous magnetism. In this context, the mechanisms underlying fusion between the lipid membranes near the critical point, which manifests interactions between individual 2-D Ising entities, may hold the key to insights into 2-D ferromagnetism.

Before concluding the study, we shall further remark on the phase behavior of the ULVs used in the current study, due to lingering concerns in three aspects: (1) a vesicle-to-vesicle variation in lipid composition; (2) discrepancy in phase behavior between the smaller ULVs adopted here (~70 nm in diameter) and larger ULVs (e.g., giant unilamellar vesicles or GUVs) treated as benchmarks; and (3) the absence of critical behaviors in the relatively small ULVs. The first concern is legitimate since a compositional variation among ULVs might lead to a variation in phase behavior and thus complicates the interpretation of the fusion assay. Though intrinsically inevitable, any compositional variation would have been insignificant, and thus each of the ULVs is expected to have been compositionally similar on average. This argument is supported by an earlier study where small-angle neutron scattering was employed to probe liquid-liquid phase separation for 4-component ULVs of ~60 nm [24]. For the scattering signals from the ULVs to be observable, the scattering power per unit volume—the scattering length density (SLD) of the ULVs—must differ from that of the surrounding solvent (here, a mixture of H_2_O and D_2_O) such that the contrasts in SLD are present. In the cited study, H_2_O and D_2_O were mixed in such a ratio that the solvent’s SLD matched that of the ULVs and led to no observable scattering signal from the ULVs when each of the ULVs was compositionally similar and exhibited no phase separation. It was reported there that the scattering signals from the ULVs could and could not be observed when the ULVs were below and above the critical temperature, i.e., with and without the phase separation, respectively. This observation proved not only that liquid-liquid phase separation exists on the nanoscopic scale but also that each of the ULVs was compositionally similar. Indeed, the scattering signals from the ULVs would have never become unobservable upon a temperature change, were the ULVs compositionally diverse. Since being prepared with a similar protocol, the ULVs used in the current study should not be meaningfully different from those of the cited study in this respect. The cited study also found that the ULVs of ~60 nm displayed a phase behavior qualitatively similar to that of the identically composed GUVs, which may ease the second concern pointed out above. The third concern arises from the suspicion that the relatively small dimension of the ULVs is not conducive to critical behaviors. However, this concern may not be warranted. Even with the relatively small dimension, the ULVs still accommodated more than 1.5 × 10^4^ lipid molecules, a number in the same order of magnitude as the numbers of spins in the Ising models of the Monte Carlo simulations where critical phenomena were studied [32,33]. (As mentioned in the Introduction, lipid membranes exhibit the same critical exponents, and thus comparable critical behaviors, as the 2-D Ising models.) In fact, an earlier study has experimentally confirmed the presence of critical phenomena for ULVs of ~90 nm by scrutinizing lipid membrane dynamics around the critical temperature with an inelastic neutron scattering technique [34]. All these arguments are expected to address the three concerns and further lend credibility to the conclusion drawn from the current study. Nevertheless, readers should be noted that the relatively small size of the ULVs might still exert confinement effects on the membranes, although these effects are not expected to considerably change the comparison among the ULVs of differing compositions since the ULVs should equally experience the confinement effects due the similarity in their sizes.

## 4. Conclusions

In summary, we employed fluorescence spectroscopy and SAXS to investigate how critical behaviors of lipid membranes, as a 2-D Ising system, are correlated with their intermembrane interaction that is manifested as membrane fusion. When the membranes were laterally heterogeneous, the increase in Chol, which drove the membranes toward the miscibility critical point, enhanced membrane fusion. This is likely due to the elevation in the membrane’s tendency of curving or the propagation of the *L_d_* − *L_o_* phase boundary in the membrane or both. On the other hand, driving the homogeneous membranes to the critical point by raising DSPC barely changed the fusion extent; however, fusion was considerably promoted once the critical point was reached and passed. This is inferred to arise from the acts of two competing factors: critical fluctuations and membrane rigidification, which might impede and promote fusion, respectively. Implications for the association between the critical fluctuations and intermembrane interaction are intriguing. Biologically, the association raises the possibility that cells regulate fusion by compositionally fine-tuning the cell membranes around the critical point. Physically, exploring the mechanisms underlying the association may provide insights into other 2-D Ising systems, including 2-D ferromagnets, whose stacking number dictates their magnetism. It is therefore of great interest to develop a theoretical framework that accommodates the association between in-plane critical behaviors and out-of-plane interactions for 2-D Ising systems in general.

## Figures and Tables

**Table 1:** Lipid compositions for the sample series used in this study.

	Composition (DOPC/DSPC/Chol, mol%)
Heterogeneous membranes	46/33/21 43/29/28 39/26/35 37/23/40 (Critical point)
Homogeneous membranes	60/0/40 49/11/40 39/21/40 37/23/40 (Critical point) 33/25/42 14/44/42 0/58/42

## Data Availability

The data will be available upon request for academic use.
